# Circadian Influences on Chemotherapy Efficacy in a Mouse Model of Brain Metastases of Breast Cancer

**DOI:** 10.3389/fonc.2021.752331

**Published:** 2021-12-09

**Authors:** William H. Walker, Samuel A. Sprowls, Jacob R. Bumgarner, Jennifer A. Liu, O. Hecmarie Meléndez-Fernández, James C. Walton, Paul R. Lockman, A. Courtney DeVries, Randy J. Nelson

**Affiliations:** ^1^ Department of Neuroscience, Rockefeller Neuroscience Institute, West Virginia University, Morgantown, WV, United States; ^2^ Department of Pharmaceutical Sciences, West Virginia University, Morgantown, WV, United States; ^3^ WVU Cancer Institute, West Virginia University, Morgantown, WV, United States; ^4^ Department of Medicine, Division of Oncology/Hematology, West Virginia University, Morgantown, WV, United States

**Keywords:** circadian rhythm, chronotherapeutic, blood-brain barrier, breast cancer, brain metastases

## Abstract

Chemotherapy is more effective in the treatment of peripheral tumors than brain metastases, likely reflecting the reduced ability of chemotherapy to cross the blood-brain barrier (BBB) and blood-tumor barrier at efficacious concentrations. Recent studies demonstrate circadian regulation of the BBB. Thus, we predicted that optimally timed chemotherapy would increase anti-tumor efficacy in a model of brain metastases of breast cancer (BMBC). First, we characterized novel daily alterations in BBB permeability to a commonly used chemotherapeutic, ^14^C-paclitaxel, within BMBC following injections given at four time points across the day. Peak and trough ^14^C-paclitaxel concentrations within BMBC occurred during the mid-dark phase and at the beginning of the light phase, respectively. Notably, chemotherapy injections during the dark phase increased cell death within BMBC and delayed onset of neurological symptoms relative to injections during the light phase. These data provide strong evidence for the beneficial effects of chrono-chemotherapy for the treatment of BMBC.

## Introduction

Physiology and behavior are optimally regulated in most organisms *via* circadian clocks. In mammals, the suprachiasmatic nucleus (SCN) of the hypothalamus is the master circadian clock and is responsible for maintaining proper synchronization of physiology and behavior with the external 24-hour day (1). Notably, circadian clocks are not restricted to the brain and are ubiquitously located throughout peripheral tissues, where they maintain synchronization of resident biological processes *via* humoral and neural signals from the SCN (2–6). Recent studies report a circadian regulation of blood-brain barrier (BBB) permeability in *Drosophila*, and demonstrate that nighttime administration of an anti-epileptic drug is more effective at treating seizures in *Drosophila* (7). In addition, circadian regulation of BBB permeability has been demonstrated in rodents (8, 9) and studies demonstrate one of the core clock genes, Bmal1, are necessary for optimal functioning of the BBB (10). However, it remains to be determined whether there are daily alterations in BBB permeability to chemotherapeutics and whether this may have functional relevance to disease treatment.

In general, chemotherapy is more effective in the treatment of peripheral tumors than brain metastases (11, 12), likely reflecting the reduced ability of chemotherapy to cross the BBB and/or blood tumor barrier (BTB) and accumulate at efficacious concentrations (13). Rodent studies demonstrate that uptake of chemotherapy within brain metastases of breast cancer (BMBC) is less than 15% of uptake in other tissues or peripheral metastases (13). Thus, cytotoxic concentrations of chemotherapy are observed only in a small subset (~10%) of brain metastases (13). Optimal treatment of brain metastases is becoming increasingly important due to the recent upward trend in incidence (14–17), and the extremely low survival rates among patients with BMBC (five-year survival <10%) (18). Indeed, because of advances in the treatment of localized breast cancer, and thus, prolonged survival of breast cancer patients, the incidence rates of subsequent BMBC are increasing (16–18); over the past two decades, estimates of BMBC incidence rates have nearly doubled from 10-15% (19) to 25-34% (20).

Because of circadian regulation of most physiological processes, efforts have been made to optimally time treatment for several diseases to improve treatment outcome while minimizing adverse effects (21–25).Chrono-chemotherapy takes advantage of predictable circadian rhythms in physiological processes to optimally time chemotherapy administration for the purpose of increasing anti-tumor efficacy and reducing adverse side effects. It is efficacious in diverse types of peripheral cancer, including bladder, ovarian, kidney, and colorectal cancers (26–30). Additionally, a recent retrospective study has demonstrated that chrono-chemotherapy may be a viable treatment option for glioblastoma (31). Nonetheless, to date, a critical gap in knowledge remains; no study has examined whether temporal alterations in chemotherapy administration can improve treatment for brain metastases.

Therefore, given (1) the demonstrated increase in anti-tumor efficacy of chrono-chemotherapy, (2) the recent revelation of circadian control of blood brain barrier permeability *via* alterations in efflux transporter function, primarily P-glycoprotein (Pgp) (7, 29, 30), and (3) that one of the most commonly prescribed drugs for the treatment of BMBC, paclitaxel (Taxol), is a substrate for Pgp transporters at the BBB (32–34), we sought to determine whether optimal timing of chemotherapy administration increases anti-tumor efficacy in a model of BMBC. We hypothesized that circadian control of the BBB/BTB alters permeability to chemotherapeutic agents. Further, we predicted that optimal timing of chemotherapy would increase anti-tumor efficacy and prolong the time until onset of neurological symptoms.

## Methods

### Experiment 1- Characterization of Daily Alterations in Paclitaxel Concentrations Within BMBC

All procedures and experiments were approved by the West Virginia University Institutional Animal Care and Use Committee (Protocol# 1801012111). Adult (>8 weeks) female NU/NU mice were purchased from Charles River Laboratories and allowed one week to acclimate prior to any experimental manipulation. Mice were provided *ad libitum* access to food (Teklad #2018) and reverse osmosis water and maintained group housed (4/cage) on a 14:10 light/dark (LD) cycle, with lights on at 0500 h. Following one week of acclimation, mice were ovariectomized to eliminate the potential confound of acute ovarian failure in response to chemotherapy administration (35). After a two-week recovery, mice received a 100 µl intracardiac injection (into the left ventricle) of the human brain seeking Her2+ breast carcinoma cell line, JIMT-1BR3-GFP-Luc (1.75x10^5^ cells) (36). Next, to confirm proper intracardiac injections, mice were injected with 150 mg/kg XenoLight D-Luciferin (Perkin Elmer; Waltham, MA) and imaged 10 minutes later (based on kinetic curve of luminescence expression for this cell line). Proper injections, indicated by luminescence confined to the brain and kidney, were confirmed *via* bioluminescence imaging (IVIS Spectrum CT, Perkin Elmer; Waltham, MA). Luminescence in the lungs suggested a miss of the left ventricle and the corresponding animals were euthanized. Following 29 days of tumor growth, mice were imaged *via* bioluminescence (IVIS Spectrum CT, Perkin Elmer; Waltham, MA) to determine the presence of brain metastases. Mice with brain metastases (>1.5e^5^ brain ROI average radiance p/s/cm²/sr) were randomly assigned to treatment groups and received a 100μl intravenous injection of ^14^C-paclitaxel (10 μCi per animal, 0.157 mg Taxol; Moravek, Brea, CA) at one of four time points (zeitgeber time (ZT) 0 (lights on), ZT5, ZT14 (lights off), ZT17). Note, intravenous tail vein injections during lights off (ZT14 and ZT17) were performed in the dark under low level red illumination. Three animals (1 from ZT14 and 2 from ZT17) were excluded from analysis due to failed tail vein injections under red light. Two hours after the injections a submandibular blood sample was obtained, the mice were perfused with saline for 30 seconds. Following perfusion, the brains were quickly removed and flash frozen in isopentane. Using a cryostat, brains were sectioned into four series (20 μm per section) and mounted onto slides (four sections per slide). One series was placed in autoradiography cassettes (Amersham Biosciences; Little Chalfont, UK) with a super resolution phosphor imaging screen (GE Healthcare; Chicago, IL) and tissue-calibrated ^14^C standards (American Radiolabeled Chemicals, Inc; St. Louis, MO) for three weeks. After three weeks of development, phosphor screens were scanned using a Typhoon phosphor imager (GE Healthcare; Chicago, IL). ^14^C-paclitaxel concentration within individual brain metastases was determined using MCID software (InterFocus Ltd; Cambridge, UK) as previously described (13) and detailed below.

### Experiment 2- Characterization of Onset of Neurological Symptoms and Sleep

Adult (>8 weeks) female NU/NU mice were purchased from Charles River Laboratories acclimated, maintained singly housed, and ovariectomized as previously described. After a two week recovery, mice received a 100 µl intracardiac injection of the human cell line, JIMT-1BR3-GFP-Luc (1.75x10^5^ cells), into the left ventricle; proper injections were confirmed as previously described. Following 14 days a tumor growth, mice were randomly assigned to treatment groups, ZT0 or ZT17, and received once a week intravenous injections (2 weeks total; tail vein) of paclitaxel (13 mg/kg) corresponding to either the peak or trough of BBB/BTB permeability (determined in Exp.1). The chosen dose of paclitaxel was 50% of the weekly human equivalent dose recommended by the National Comprehensive Cancer Network treatment guidelines for the treatment of metastatic HER2+ breast cancer. Human equivalent dose was calculated as previously reported (37). Mice were monitored daily for onset of neurological symptoms (defined as the presence of head tilt, motor ataxia, or circling behavior). Upon onset of neurological symptoms, mice were imaged *via* bioluminescence as previously described, perfused with saline for 30 seconds, and the brains were quickly removed and flash frozen. A subset of animals n=8/group were housed in PiezoSleep mouse behavioral tracking system cages (Signal Solutions LLC, Lexington, KY) prior to intracardiac injections and continued until study completion. Sleep was analyzed using the default scoring in SleepStats v2.18 (Signal Solutions LLC, Lexington, KY). Monitoring and determination of neurological symptoms were conducted by one experimenter who was unaware of experimental group assignment and verified by a second experimenter who also was unaware of experimental group assignment. One mouse had an acute adverse reaction (within 24 h) to the chemotherapy administration and was not included in the analysis. To rule out possible effects of chemotherapy alone on presentation of neurological symptoms, a second cohort of mice received a 100 µl intracardiac injection of vehicle (DMEM; Gibco, Waltham, MA), into the left ventricle (no tumor cells). Fourteen days later, the mice were randomly assigned to treatment groups, ZT0 or ZT17, and received an injection of paclitaxel (13 mg/kg; IV). A second injection was given 7 days later. Mice were monitored daily for onset of neurological symptoms (defined as the presence of head tilt, motor ataxia, or circling behavior). These mice were continuously monitored until the completion of the study at 45 days. Note, similar to the previous cohort, one mouse had an acute adverse reaction to the chemotherapy administration (within 24 h) and was not included in the analysis).

### Experiment 3- Characterization of Tumor Cell Death

Adult (>8 weeks) female NU/NU mice were purchased from Charles River Laboratories acclimated, maintained singly housed, and ovariectomized as previously described. After a two week recovery, mice received a 100 µl intracardiac injection of the human cell line, JIMT-1BR3-GFP-Luc (1.75x10^5^ cells), into the left ventricle. Proper injections were determined *via* bioluminescence imaging (IVIS Spectrum CT, Perkin Elmer; Waltham, MA) as previously described. Following 14 days of tumor growth, mice were randomly assigned to treatment groups, ZT0 or ZT17, and received once a week, intravenous injection (2 weeks total; tail vein) of paclitaxel (13 mg/kg) corresponding to either the peak or trough of BBB permeability (determined in Exp.1). Eight hours following the second chemotherapy dose (13, 38), mice were perfused with 4% paraformaldehyde for 30 seconds. Following perfusion, the brains were extracted, dropped fixed in 4% paraformaldehyde overnight, and placed in 30% sucrose solution the next day. Brains were sectioned into four series (20 μm per section) and stained *via* free-floating immunofluorescence for cleaved caspase-3 and cytokeratin 5/8. The amount of cell death within BMBC was calculated by determining the percent area of double-labeled cells (AC3+ Cytokeratin 5/8/GFP+) within a tumor using the triangle threshold on Fiji (39, 40).

### Experiment 4- Characterization of Onset of Neurological Symptoms in Murine Model of BMBC (4T1-BR5)

Adult (>8 weeks) female NU/NU mice were purchased from Charles River Laboratories acclimated, maintained singly housed, and ovariectomized as previously described. After a two week recovery, mice received a 100 µl intracardiac injection of the murine brain seeking breast carcinoma cell line, 4T1-BR5-Luc (1.75x10^5^ cells), into the left ventricle; proper injections were confirmed as previously described. Following 5 days a tumor growth, mice were randomly assigned to treatment groups, ZT0 or ZT17, and received once a week intravenous injections (2 weeks total; tail vein) of paclitaxel (13 mg/kg) corresponding to either the peak or trough of BBB/BTB permeability (determined in Exp.1). Note, a subset of mice received only one injection of paclitaxel on Day 5 due to reaching early removal criteria (ERC; i.e., the onset of neurological symptoms) prior to the second chemotherapy injection (Day 12). Mice were monitored daily for onset of neurological symptoms (defined as the presence of head tilt, motor ataxia, or circling behavior). Upon onset of neurological symptoms, mice were imaged *via* bioluminescence as previously described, perfused with saline for 30 seconds, and the brains were quickly removed and flash frozen. Monitoring and determination of neurological symptoms were conducted by one experimenter who was unaware of experimental group assignment and verified by a second experimenter who also was unaware of experimental group assignment.

### Ovariectomy Procedure

Mice were fed a diet containing the non-steroidal anti-inflammatory drug carprofen 24 hours prior to and 48 hours following surgery for analgesia. In preparation for surgery, mice were anesthetized using 2% isoflurane and placed prone. A midline skin incision (1 cm) was made allowing for the movement and visualization of each ovarian fat pad. A small (0.5 cm) incision was made directly over the ovarian fat pad and the fat pad was withdrawn to allow for visualization of the ovary and uterine horn. Each ovary was removed from the uterine horn *via* cauterization and the uterine horn was returned into the abdominal cavity. The muscle was sutured and the contralateral ovary was removed as described. Following removal of both ovaries, the skin incision was closed using tissue glue.

### MCID Analysis

In short, phosphor images were converted to color-coded images based on ^14^C tissue concentrations, using MCID software (InterFocus Ltd; Cambridge, UK). Next, all sections were stained *via* cresyl violet staining to visualize brain metastases. Following cresyl violet staining, all brain sections were imaged at 2X (Keyence BZ-X710; Itasca, IL) and overlaid with its corresponding color-coded phosphor image using Adobe Photoshop (Adobe Inc.; San Jose, CA). The overlaid images were then loaded into MCID and the channels were linked. Tumors were visualized and outlined on the cresyl violet image, while simultaneously, MCID overlaid the corresponding outline onto the color-coded phosphor image. Using the default threshold algorithm in MCID, neoplastic tissue within the outlined shape was highlighted and the amount of ^14^C was calculated using the standard curve (calculated in MCID based on ^14^C standards) for that animal. Cresyl violet sections in which the tumor could not be clearly defined were not included in analysis; two animals were excluded from analysis due to an error in cresyl violet staining and thus, an inability to localize the tumors. All MCID analyses and image overlays were completed by experimenters unaware of treatment groups.

### Cresyl Violet Staining

Slides were placed into distilled water for 3 minutes and then dipped 5 times in each of the following solutions: (1) 70% EtOH, (2) 70% EtOH, (3) 95% EtOH, (4) 95% EtOH, (5) 100% EtOH, (6) 100% EtOH, (7) 100% Xylene, (8) 100% Xylene, (9) 100% Xylene. Once finished with the last xylene wash, sections were then dipped 5 times in each of the solutions in the reverse order (9 to 1) and concluding in five dips in water. Following the water dips, slides were placed in 0.1% cresyl violet acetate solution for 4 minutes. Excess stain was rinsed off in water and sections were dipped in 70% EtOH six times. Next, slides were dipped 5 times and alternated between 95% EtOH and 95% EtOH+Glacial acetic acid. Finally, slides were dipped 5 times in 100% EtOH and 100% xylene and cover slipped with Permount.

### Immunofluorescence and Fiji Analysis

Double labeled immunofluorescence for cleaved caspase-3 (AC3) and cytokeratin 5/8 occurred on 20 μm free floating sections. Sections were washed 5 times for 5 minutes in 1XPBS. After washing, sections were placed in 0.05 M sodium citrate (pH 6.2) for 30 minutes. Next, slides were placed in a blocking solution containing 1XPBS, 0.1% Triton-X100, 2.5% NDS, and 2.5% NGS for 1 h. Following the one hour block, sections were incubated at room temperature overnight in the primary antibody cocktail (rabbit anti-cleaved caspase-3 (#9664S) 1:500, Cell Signaling Technology, Danvers, MA; mouse anti- cytokeratin 5/8 (ab9005) 1:500, Abcam, Cambridge, UK) plus blocking solution. The following day sections were washed 5 time for 5 minutes in 1XPBS and incubated in the secondary antibody cocktail (488 goat anti-mouse, 1:250, Life Technologies Corporation, Eugene, OR; 594 donkey anti-rabbit, 1:250, Life Technologies Corporation, Eugene, OR) plus blocking solution for 2 h. Sections were washed again and cover slipped with VectaShield + DAPI mounting media (Vector Labs, Burlingame, CA). Next, twelve brain metastases were randomly selected from each brain and imaged at 20X (Keyence BZ-X710; Itasca, IL). The randomly selected brain metastases were equally distributed throughout the brain with four brain metastases randomly selected from an anterior (Start- ~0.13 bregma), medial (~0.13- ~-1.79), and posterior (~-1.79-End) portion of the brain. Three mice did not have twelve separate brain metastases and thus all tumors possible were included. Percent area fraction (i.e., the portion of the region of interest that is above threshold and therefore positively stained) was quantified within each brain metastasis. To analyze the percent area fraction of cleaved caspase-3 staining within brain metastases, tumor images were loaded into Fiji, and a freehand selection was made around the entire tumor and nearby parenchymal area. The area outside of the selection was cleared, the channels were split into RGB (R – cleaved caspase-3, G – cytokeratin 5/8/GFP, B - DAPI), and a mask of positive cytokeratin 5/8/GFP staining was created. The cytokeratin/GFP staining mask was created by first applying an automatic threshold (“Triangle dark” algorithm) to the green channel. Next, the thresholded regions were refined by sequentially applying the Erode, Removal of Outliers (radius=3, threshold=50, which=Dark), and Dilate functions. Finally, a selection of the thresholded area was created and applied to the red channel. Using an automatic threshold (“Triangle dark” algorithm), the percent area fraction of cleaved caspase-3 staining within brain metastases (i.e., outline of positive cytokeratin/GFP staining) was calculated. All Fiji analyses were completed by an experimenter unaware of treatment groups.

### Statistical Analyses

**Figure 1:**
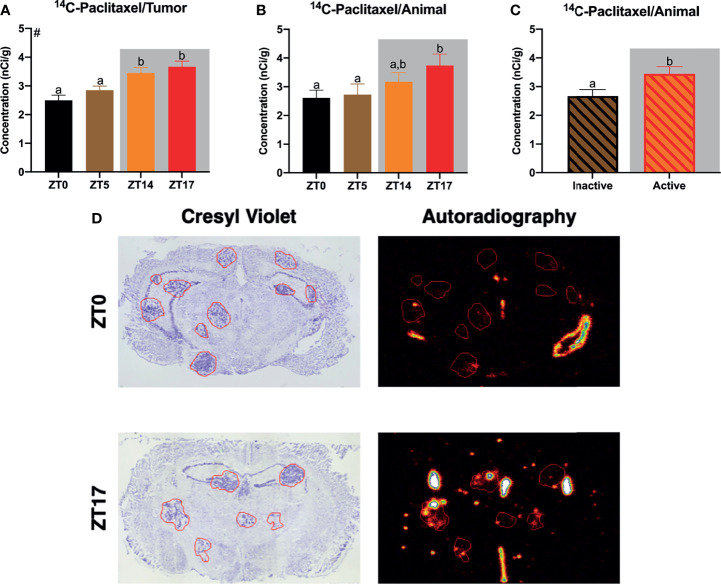
Daily alterations in ^14^C-paclitaxel within JIMT-1BR3 BMBC. **(A)** When examining all tumors independently, mice receiving paclitaxel injections at ZT14 or ZT17 demonstrated significantly increased ^14^C-paclitaxel concentrations within BMBC relative to mice receiving paclitaxel injections at ZT0 or ZT5. **(B)** When examining animal averages, planned comparisons demonstrate an increase in ^14^C-paclitaxel concentrations within BMBC at ZT17 relative to ZT0 or ZT 5. **(C)** Additionally, when combining groups and assessing animal averages based on activity, mice that received paclitaxel injections during their active phase displayed significantly increased ^14^C-paclitaxel concentrations within BMBC. **(D)** Representative cresyl violet and autoradiography images for ZT0 and ZT17. [**(A)** n= 122, 269, 185, 142 respectively; **(B)** n= 9, 9, 9, 8 respectively; **(C)** n= 18, 17 respectively]. The data are presented as mean +SEM. Graph bars that do not share a letter are statistically significantly different at p<0.05. ^#^Main effect of time of injection; **(A)** nonparametric Kruskal-Wallis test; Dunn’s multiple comparisons test **(B)** one-way ANOVA; Fisher’s LSD multiple comparisons test **(C)** unpaired *t*-test.

**Figure 2:**
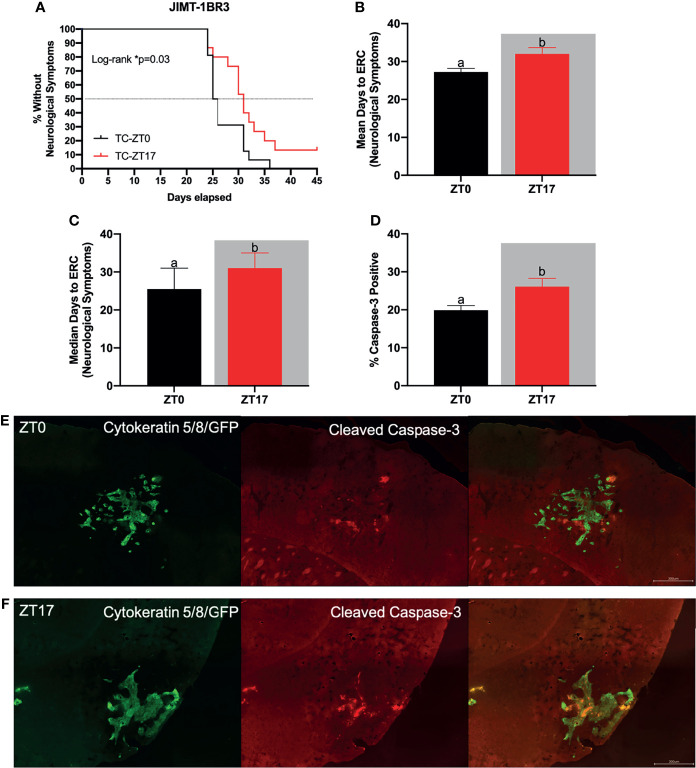
Optimal timing of chemotherapy delays onset of neurological symptoms and increases cell death within JIMT-1BR3 BMBC. **(A)** Mice harboring BMBC and receiving paclitaxel injections at ZT17 (ie TC-ZT17, n=15) demonstrated prolonged onset of neurological symptoms relative to mice harboring BMBC and receiving paclitaxel injections at ZT0 (i.e., TC-ZT0, n=16). Mice receiving paclitaxel injections alone did not display neurological deficits irrespective of administration time (i.e., C-ZT0, n=14 and C-ZT17, n=15). Additionally, mice harboring BMBC and receiving paclitaxel injections at ZT17 demonstrated significantly prolonged **(B)** mean and **(C)** median number of days until the onset of neurological symptoms and significantly increased **(D)** cell death with BMBC relative to ZT0. **(E, F)** Representative images of cell death within BMBC at ZT0 and ZT17. For negative controls see **Supplemental Figures 1** and **2**. Green- cytokeratin 5/8/GFP, Red- cleaved caspase-3. (B. n= 16,15 respectively; **(C)** n= 16,15 respectively; **(D)** n= 11, 11 respectively). The data are presented as mean +SEM for **(B, D)** and median +95% CI for **(C)**. Graph bars that do not share a letter are statistically significantly different at p<0.05. * Log-rank p<0.05. **(A)** Log-rank test **(B)** unpaired *t*-test **(C)** Mann Whitney test **(D)** unpaired *t*-test.

**Figure 3:**
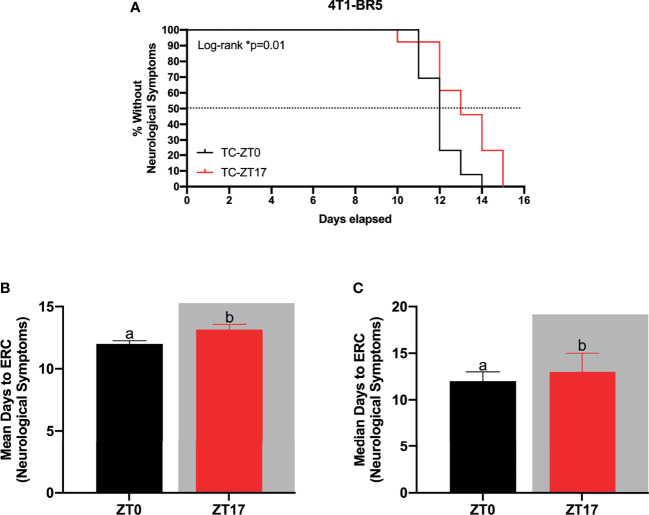
Optimal timing of chemotherapy delays onset of neurological symptoms in 4T1-BR5 murine model of BMBC. **(A)** Administration of paclitaxel at ZT17 (i.e., TC-ZT17, n=13) prolonged the onset of neurological symptoms relative to mice harboring BMBC and receiving paclitaxel injections at ZT0 (i.e., TC-ZT0, n=13). Additionally, mice harboring BMBC and receiving paclitaxel injections at ZT17 demonstrated significantly prolonged **(B)** mean and **(C)** median number of days until the onset of neurological symptoms relative to ZT0. The data are presented as mean +SEM for **(B)** and median +95% CI for **(C)**. Graph bars that do not share a letter are statistically significantly different at p<0.05. * Log-rank p<0.05. **(A)** Log-rank test **(B)** unpaired *t*-test **(C)** Mann Whitney test.

**Figure 4:**
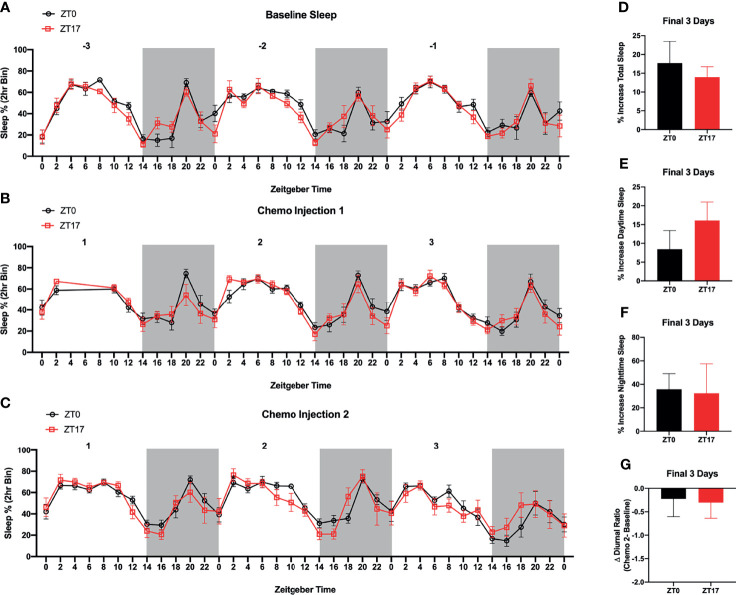
Timing of Paclitaxel Administration Does Not Alter Sleep/Wake States in Mice Harboring JIMT-1BR3 BMBC. Sleep/Wake states did not significantly differ between **(A)** baseline, **(B)** three days following the first paclitaxel injection, or **(C)** three days following the second paclitaxel injection. There was an unexpected power outage following the first paclitaxel injection, day 1 ZT4-8. Additionally, whereas in both groups there was an increase in **(D)** total sleep, **(E)** sleep during the daytime, and **(F)** sleep during the nighttime in the three days following the second paclitaxel injection relative to baseline sleep, these effects were not specific to one group. Further, there was no change in the **(G)** diurnal ratio between groups. **(A–C)**. n= 8,7 respectively; **(D–F)**. n= 8,6 respectively; **(G)** n=7,5 respectively). The data are presented as mean +SEM. **(A, B)** Two-way repeated measures ANOVA; Fisher’s LSD multiple comparisons test. **(C)** Mixed effect analysis; Fisher’s LSD multiple comparisons test. **(D–G)** unpaired *t*-test.

Prior to any statistical analysis outliers were identified and removed using the Grubbs’ test (41, 42). Note, for **Figures 1B, C** outliers were identified and removed at the level of the tumors, not animal averages. This was chosen as an outlier in tumor concentrations in ^14^C-paclitaxel could dramatically alter animal averages. Student’s *t*-tests were used to analyze **Figures 1C**, **2B, D**, **3B**, and **4D–G**. Median days to early removal criteria (**Figures 2C** and **3C**) was analyzed *via* Mann-Whitney test. A one-way ANOVA was used to analyze **Figure 1B**. *Post-hoc* analysis was performed using Fisher’s LSD tests. The data in **Figure 1A** failed to meet the homoscedasticity assumption of an ANOVA (i.e., Brown-Forsythe Test p=0.0132). Therefore, a nonparametric Kruskal-Wallis test was performed. *Post-hoc* analysis was performed using Dunn’s multiple comparisons test. Additionally, a two-way repeated measures ANOVA was used to analyze the sleep data (**Figures 4A, B**). A mixed effect analysis was used to analyze the sleep data for **Figure 4C**, as one animal’s sensor malfunctioned, resulting in data from Day 1 ZT16-Day3 to be excluded for that animal. *Post-hoc* analyses were performed using a Fisher’s LSD test. Finally, a Log-rank test was used to compare ERC data, **Figures 2A** and **3A**. Comparisons were only made between tumor groups (i.e., TC-ZT0 and TC-ZT17). For all mean comparisons, p<0.05 was considered statistically significant. All statistical analyses were performed using GraphPad Prism 8.4 software.

## Results

### Characterization of Daily Alterations in Paclitaxel Concentrations Within BMBC

We first sought to determine whether timing of ^14^C-paclitaxel administration altered chemotherapy concentrations within BMBC. Indeed, when examining all tumors independently, there was a main effect of time of day in ^14^C-paclitaxel concentrations within the lesions (H(3)=33.03, p<0.0001). Specifically, mice that received injections at ZT 14 or ZT17 (during the dark period) demonstrated increased ^14^C-paclitaxel concentrations within BMBC relative to both ZT0 and ZT5 (during the light period; **Figure 1A**; p<0.05; Dunn’s multiple comparisons). When examining means (at the level of animal) there was not a significant effect of time of day (**Figure 1B** F_3,31_ = 2.182; p>0.05). However, planned comparisons demonstrated significantly increased ^14^C-paclitaxel concentrations in mice that received injections at ZT17 relative to mice that received chemotherapy injections at ZT0 or ZT5 (**Figure 1B**; p<0.05; Fisher’s LSD). There was a significant effect of time of day when comparing means between mice injected during their inactive (light) or active (dark) phase. Specifically, mice receiving chemotherapy injections during the dark phase demonstrated an ~30% increase in ^14^C-paclitaxel concentrations relative to mice that were administered chemotherapy during the light phase (**Figures 1C, D**; *t*=2.292; p<0.05).

### Characterization of Onset of Neurological Symptoms and Cell Death

Next, we assessed the functional significance of increased ^14^C-paclitaxel concentrations within BMBC when chemotherapy was administered during the dark phase by monitoring the onset of neurological symptoms and cell death within the lesions. Tumor-bearing mice that received chemotherapy injections at ZT17 demonstrated significantly delayed onset of neurological symptoms when compared to tumor-bearing mice that received chemotherapy injections at ZT0 (**Figure 2A**.; χ^2^ = 4.629; p<0.05); both mean and median number of days until the onset of neurological symptoms was increased among mice treated at ZT17 relative to ZT0 (32 vs. 27.25 and 31 vs. 25.5, respectively) (**Figures 2B, C**; *t*=2.544 and U=70, respectively; p<0.05 for both comparisons). The appearance of neurological symptoms was not directly related to chemotherapy treatment; in the absence of BMBC, neurological symptoms were not recorded in any animals, regardless of time of chemotherapy injection (**Figure 2A**). To determine how altering timing of chemotherapy injections delayed onset of neurological symptoms, we assessed the effects of timing of chemotherapy administration on cell death within BMBC. Mice that received injections of paclitaxel at ZT17 demonstrated a significant increase in cleaved caspase-3 staining within BMBC relative to mice that received injections at ZT0. Specifically, cleaved caspase-3 within lesions increased ~30% in mice that received injections at ZT17 (**Figures 2D–F**; *t*=2.518; p<0.05).

To verify the functional significance of increased ^14^C-paclitaxel concentrations within BMBC when chemotherapy was administered during the dark phase, a second experiment using the murine brain seeking cell line 4T1-BR5 was performed. Mice harboring BMBC and receiving paclitaxel injections at ZT17 demonstrated significantly delayed onset of neurological symptoms when compared to tumor-bearing mice that received chemotherapy injections at ZT0 (**Figure 3A**; χ^2^ = 5.083; p<0.05). Notably, even in a more aggressive tumor model (i.e., 4T1-BR5; **Figure 3A** vs JIMT-1BR3; **Figure 2A**), administration of paclitaxel at ZT17 significantly increased both mean and median number of days until the onset of neurological symptoms (**Figures 3B, C**; *t*=2.347 and U=42.50, respectively; p<0.05 for both comparisons).

### Characterization of Sleep

As a readout of circadian rhythms, we assessed sleep/wake cycles in mice harboring BMBC and receiving paclitaxel injections at ZT0 or ZT17. There was no effect of timing of chemotherapy injections or interaction between timing of chemotherapy injection and time of day on sleep/wake cycles, three days prior to JIMT-1 injections (i.e., baseline) (**Figure 4A**; F_1,13 _= 1.104 and F_36,468 _= 0.9611 respectively; p>0.05), three days following the first paclitaxel injection (**Figure 4B**; F_1,13 _= 0.6775 and F_33,429 _= 0.8421 respectively; p>0.05), or three days following the second paclitaxel injection (**Figure 4C;** F_1,13 _= 0.1174 and F_36,439 _= 0.9398 respectively; p>0.05). As expected, there was a main effect of time of day on sleep wake states (**Figures 4A–C**; F_5.679,73.83 _= 17.69, F_5.740,74.61 _= 16.24, F_5.164,62.97 _= 12.44 respectively; p<0.0001). Additionally, although in both groups there was an increase in total sleep, sleep during the daytime, and sleep during the nighttime in the three days following the second paclitaxel injection relative to baseline sleep, these effects were not altered by timing of chemotherapy administration (**Figures 4D–F**; p>0.05). Further, there were no significant effects of time of chemotherapy administration when assessing changes in the diurnal ratio of sleep (i.e., daytime/nighttime sleep) (**Figure 4G**; p>0.05).

## Discussion

Given (1) the increase in anti-tumor efficacy of chrono-chemotherapy for the treatment of peripheral tumors, (2) the recent revelation of circadian control of blood brain barrier permeability *via* alterations in efflux transporter function, primarily P-glycoprotein (Pgp) (7, 29, 30), and (3) that one of the most commonly prescribed drugs for the treatment of BMBC, paclitaxel (Taxol), is a substrate for Pgp transporters at the BBB (32–34), the current study sought to determine whether chrono-chemotherapy was a viable treatment strategy to improve the treatment of BMBC.

First, we demonstrate daily alterations in chemotherapy uptake within BMBC; by altering only the timing of ^14^C-paclitaxel administration, paclitaxel concentrations changed significantly within the lesions (**Figures 1A–C**). Specifically, peak concentrations were achieved in mice receiving chemotherapy injections at ZT17 whereas trough concentrations were displayed in mice receiving chemotherapy injections at ZT0 (~45% increase). Notably, these effects remain consistent whether considering each tumor as an independent data point or generating a mean of all tumors within an animal (**Figures 1A, B**). Previous studies have reported daily alterations in blood brain barrier (BBB) permeability to xenobiotic substrates (43). Indeed, a recent study in *Drosophila* demonstrated circadian regulation of BBB permeability to xenobiotic substrates *via* daily alterations in the activity of an efflux transporter homologous to Pgp (7). Additionally, the authors determined functional significance by demonstrating that optimally timed administration of the anti-epileptic drug, phenytoin, was more effective at treating a *Drosophila* seizure model (7). Similar to the previous study, additional studies in rats have demonstrated daily alterations in permeability to xenobiotic substances that are substrates for Pgp (44, 45). Notably, paclitaxel is primarily a Pgp substrate (33, 34, 46) (some evidence suggests it is also a substrate for multidrug resistance protein 2) (47) and pharmacologically or genetically impairing the function of Pgp transporters substantially increases the concentration of paclitaxel in the brains of wild type mice (33). Thus, we propose that daily fluctuations in paclitaxel concentrations within BMBC are likely due to changes in Pgp activity at the BBB/BTB across the day.

Next, we assessed the functional significance of temporally-induced variation in ^14^C-paclitaxel concentrations within BMBC by assessing the onset of neurological symptoms and tumor-associated cell death. Mice that were administered paclitaxel at ZT17, corresponding to the peak concentrations of paclitaxel within BMBC, demonstrated increased cell death and delayed onset to develop neurological symptoms relative to mice receiving paclitaxel injections at ZT0 (**Figures 2A–F**). Specifically, administration of chemotherapy at ZT17 resulted in an ~20% increase in median survival (**Figure 2**). To verify the functional significance of increased ^14^C-paclitaxel concentrations within BMBC when chemotherapy was administered during the dark phase, a second experiment using the murine brain seeking cell line 4T1-BR5 was performed. Consistent with the human brain seeking cell line, JIMT-1BR3, administration of paclitaxel at ZT17 significantly delayed the onset of neurological symptoms relative to mice receiving paclitaxel injections at ZT0 (**Figures 3A–C**). Notably, this suggests that even in a more aggressive cancer model (i.e., terminal day to ERC day 15 in 4T1-BR5 vs day 45 in JIMT-1BR3) chrono-chemotherapy can have beneficial treatment effects. Together, these data are consistent with previous studies demonstrating prolonged survival in patients receiving chrono-modulated chemotherapy regimens for ovarian cancer, colorectal cancer, and leukemia (48–51). One such phase III clinical trial in patients with peripheral ovarian cancer, demonstrated that morning doxorubicin treatment followed with evening cisplatin treatment was advantageous in reducing adverse side effects and increased the 5 year survival rate from 11% to 44% (48). Additionally, it is not surprising that administration of paclitaxel at ZT17 increases cell death within BMBC relative to administration of paclitaxel at ZT0, as paclitaxel concentrations increase by 43% (**Figure 1B**)-47% (**Figure 1A**) in mice receiving chemotherapy injections at ZT17 relative to ZT0. Indeed, previous studies have demonstrated a positive relationship between intratumoral concentrations of chemotherapy and cell death within breast cancer or BMBC (13, 52–54).

Finally, as a readout of circadian rhythms, we assessed sleep/wake cycles in mice harboring BMBC and receiving paclitaxel injections at ZT0 or ZT17. Sleep/wake cycles remained unchanged between groups during three days prior to JIMT-1 injections, three days following the first paclitaxel injection, and three days following the second paclitaxel injection (**Figures 4A–C**). In both groups, there was an expected increase in total sleep, daytime sleep, and nighttime sleep in the three days following the second paclitaxel injection relative to baseline sleep; however, these effects did not differ by timing of the chemotherapy injection (**Figures 4D–F**). Further, the diurnal ratio (i.e., ratio of daytime/nighttime sleep) remained relatively unchanged in the three days following the second paclitaxel injection relative to baseline sleep (**Figure 4G**). Previous studies have examined changes in sleep following chemotherapy administration in patients and mice and demonstrate that chemotherapy treatment can significantly alter sleep/wake states (55–57). In patients, chemotherapy treatment is associated with poor sleep quality, increased sleep fragmentation, and increased sleep/daytime sleepiness (58–60). Consistent with increased sleep in the present study, studies in rodents have demonstrated increased NREM and REM sleep and increased sleep fragmentation following a single dose of doxorubicin/cyclophosphamide chemotherapeutic cocktail (57). The authors conclude that these effects are likely due to increased pro-inflammatory cytokine signaling within the brain. To our knowledge, however, this is the first study to examine the effects of differential timing of chemotherapy administration on sleep; it is possible that the current study paradigm was not sufficiently sensitive to detect subtle changes in sleep/wake states between groups or that the presence of brain tumors within both groups masked the effects of chrono-chemotherapy on sleep. Thus, future studies should examine the effects of chrono-chemotherapy on sleep using EEG and EMG analyses and/or nonmetastatic cancer models.

### Limitations/Future Directions

Although the timing of paclitaxel administration had striking effects on accumulation of the drug within the BMBC and post-treatment survival in the current study, future studies will need to assess the generality of these observations through the examination of additional cancer types and additional chemotherapeutics. In the present study, we examined the effects of optimally timed paclitaxel for the treatment of two cancer cell lines: (1) the human brain seeking Her2+ breast carcinoma cell line, JIMT-1BR3-GFP-Luc and (2) the murine brain seeking breast carcinoma cell line, 4T1-BR5-Luc. Notably, expression of efflux transporters can change dramatically in different types of cancer and/or within cancer subtypes (i.e., different breast cancer cells) (61). Additionally, binding of chemotherapeutics to efflux transporters can vary depending on the chemotherapeutic (62). For example, paclitaxel is a Pgp substrate, whereas doxorubicin is a substrate for Pgp and BCRP (62). Therefore, future studies may need to determine the optimal timing of chemotherapy administration specific to use of chemotherapeutic and tumor efflux transporter expression. Furthermore, circadian changes in efflux transporter function may not underlie the current daily alterations in paclitaxel concentrations within BMBC. An alternative explanation may be that glymphatic/interstitial fluid clearance within the brain may underlie circadian changes in paclitaxel concentrations within BMBC. Indeed, a recent study demonstrated circadian alterations in glymphatic/interstitial fluid clearance within the brain (63). The authors demonstrate that peak clearance of interstitial fluid within the brain occur during the inactive phase in mice. This observation is consistent with the effects seen in the current study, in that peak paclitaxel concentrations within BMBC occur during the active phase, whereas trough concentrations within BMBC occur during the inactive phase in mice. Thus, future studies should determine what role circadian alterations in efflux transporter function and/or circadian alterations in interstitial fluid clearance play in the observed daily changes in paclitaxel concentrations within BMBC. Finally, the current study did not assess changes in tumor growth in response to optimally timed chemotherapy administration due to the concern that the repeated anesthesia exposures needed for *in vivo* bioluminescent imaging could disrupt the circadian rhythms of the mice, in turn undermining the precise timing required by the experimental design. Whether the prolonged survival following chemotherapy treatment at ZT 17 is associated with a concomitant reduction in tumor size is an interesting question that will need to be addressed in future studies.

In sum, the present study demonstrates daily alterations in the uptake of chemotherapy within BMBC. Mice administered chemotherapy during their active phase, primarily ZT17, displayed increased concentrations of paclitaxel within BMBC relative to mice administered chemotherapy during their inactive phase, primarily ZT0. Administration of paclitaxel at ZT17 significantly increased cell death within BMBC and delayed the onset of neurological symptoms in mice. Together, these data demonstrate that chrono-chemotherapy is a novel and potentially effective treatment strategy for BMBC, and emphasizes the importance of time of day when administering chemotherapy to patients.

## Data Availability Statement

The raw data supporting the conclusions of this article will be made available by the authors, without undue reservation.

## Ethics Statement

The animal study was reviewed and approved by West Virginia University Institutional Animal Care and Use Committee.

## Author Contributions

WWII designed, completed, analyzed all experiments and wrote the manuscript. JB designed, conducted experiments, and provided editorial comments on the paper. SS, JL, and OM-F, conducted experiments and provided editorial comments on the paper. JW designed, analyzed, and provided editorial comments on the paper. RN, AD, and PL designed experiments and wrote and edited the manuscript. All authors contributed to the article and approved the submitted version.

## Funding

The authors were supported by grants from NCI (5R01CA194924 to AD), NINDS (5R01NS092388 to RN/AD), NIGMS (P20GM121322 to PL), WVU Mylan Chair Endowment Fund (PL), NCI (F99CA253768-01 to SS), and NIGMS under award number 5U54GM104942-03.

## Author Disclaimer

The content is solely the responsibility of the authors and does not necessarily represent the official views of the National Institutes of Health.

## Conflict of Interest

The authors declare that the research was conducted in the absence of any commercial or financial relationships that could be construed as a potential conflict of interest.

## Publisher’s Note

All claims expressed in this article are solely those of the authors and do not necessarily represent those of their affiliated organizations, or those of the publisher, the editors and the reviewers. Any product that may be evaluated in this article, or claim that may be made by its manufacturer, is not guaranteed or endorsed by the publisher.

## Supplementary Material

Ensure that any supplementary material is correctly published at this link: https://www.frontiersin.org/articles/10.3389/fonc.2021.752331/full#supplementary-material
