# Supplementary material for: Circadian Influences on Chemotherapy Efficacy in a Mouse Model of Brain Metastases of Breast Cancer
Source: Front Oncol. 2021 Dec 9;11:752331. doi: 10.3389/fonc.2021.752331 (PMC8695439; doi:10.3389/fonc.2021.752331)
Supplement: Supplementary file 1 [file DataSheet_1.docx]

Supplemental Information


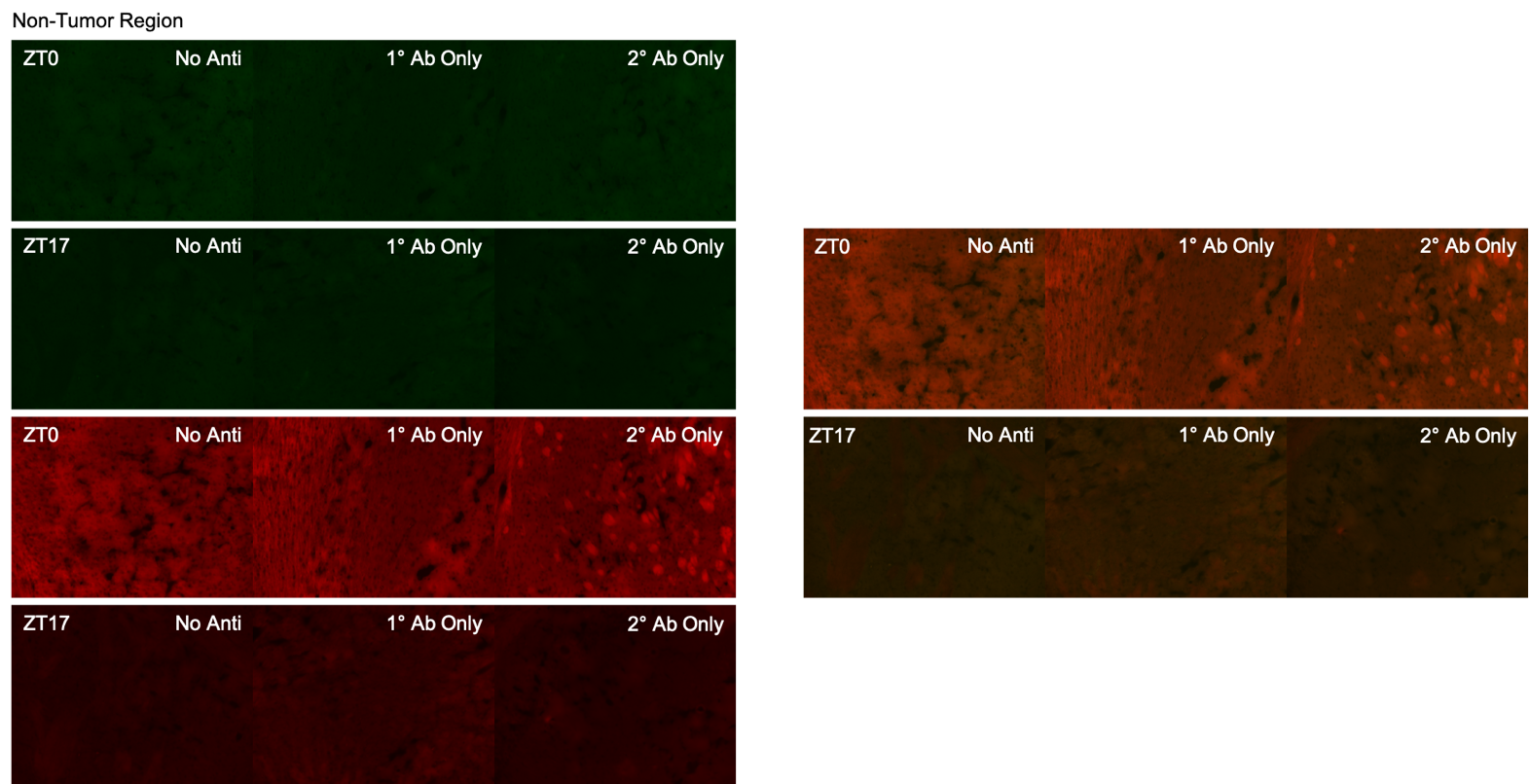


Supplemental Figure 1: Appropriate negative controls for ZT0 and ZT17 in a non-tumor region of the brain. Top two left: GFP channel only. Bottom two left: Texas Red channel only. Right two: GFP and Texas Red channels.


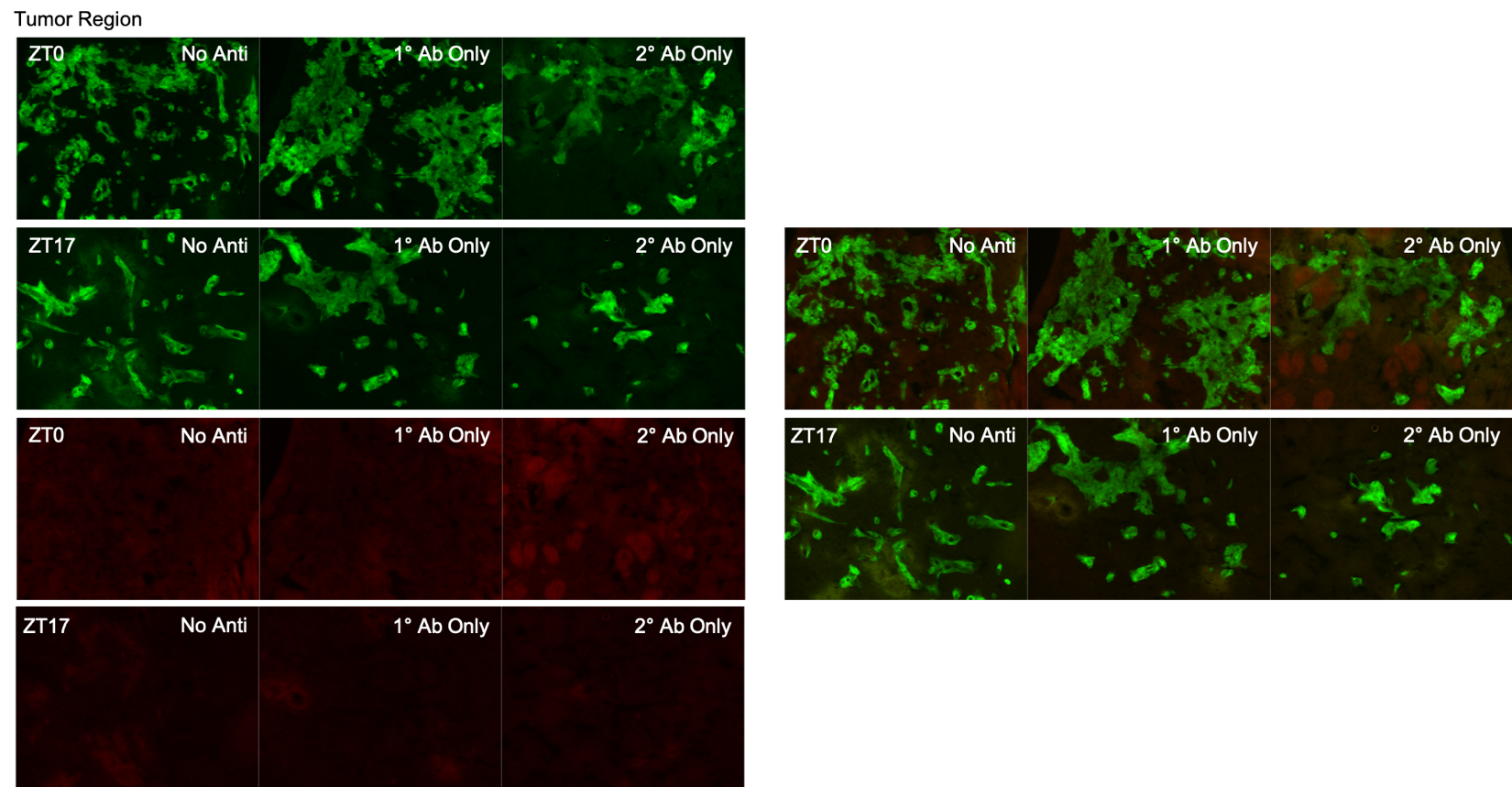


Supplemental Figure 2: Appropriate negative controls for ZT0 and ZT17 in a tumor region of the brain. Top two left: GFP channel only. Bottom two left: Texas Red channel only. Right two: GFP and Texas Red channels. Note: JIMT-1BR3 cells express GFP.
